# *Plasmodium vivax:* the potential obstacles it presents to malaria elimination and eradication

**DOI:** 10.1186/s40794-022-00185-3

**Published:** 2022-12-15

**Authors:** Kassahun Habtamu, Beyene Petros, Guiyun Yan

**Affiliations:** 1 Department of Microbial, Cellular & Molecular Biology, Addis Ababa University, Addis Ababa, Ethiopia; 2 Menelik II Medical & Health Science College, Addis Ababa, Ethiopia; 3 Program in Public Health, University of California at Irvine, Irvine, CA 92697 USA

**Keywords:** *P. vivax*, *P. falciparum*, Hypnozoite, Primaquine, G6PD, CYP 2D6, Tafenoquine

## Abstract

Initiatives to eradicate malaria have a good impact on *P. falciparum* malaria worldwide. *P. vivax*, however, still presents significant difficulties. This is due to its unique biological traits, which, in comparison to *P. falciparum*, pose serious challenges for malaria elimination approaches. *P. vivax's* numerous distinctive characteristics and its ability to live for weeks to years in liver cells in its hypnozoite form, which may elude the human immune system and blood-stage therapy and offer protection during mosquito-free seasons. Many malaria patients are not fully treated because of contraindications to primaquine use in pregnant and nursing women and are still vulnerable to *P. vivax* relapses, although there are medications that could radical cure *P. vivax*. Additionally, due to CYP2D6's highly variable genetic polymorphism, the pharmacokinetics of primaquine may be impacted. Due to their inability to metabolize PQ, some CYP2D6 polymorphism alleles can cause patients to not respond to treatment. Tafenoquine offers a radical treatment in a single dose that overcomes the potentially serious problem of poor adherence to daily primaquine. Despite this benefit, hemolysis of the early erythrocytes continues in individuals with G6PD deficiency until all susceptible cells have been eliminated. Field techniques such as microscopy or rapid diagnostic tests (RDTs) miss the large number of submicroscopic and/or asymptomatic infections brought on by reticulocyte tropism and the low parasitemia levels that accompany it. Moreover, *P. vivax* gametocytes grow more quickly and are much more prevalent in the bloodstream*. P. vivax* populations also have a great deal of genetic variation throughout their genome, which ensures evolutionary fitness and boosts adaptation potential. Furthermore, *P. vivax* fully develops in the mosquito faster than *P. falciparum*. These characteristics contribute to parasite reservoirs in the human population and facilitate faster transmission. Overall, no genuine chance of eradication is predicted in the next few years unless new tools for lowering malaria transmission are developed (i.e., malaria elimination and eradication). The challenging characteristics of *P. vivax* that impede the elimination and eradication of malaria are thus discussed in this article.

## Introduction

The protozoan parasites of the genus Plasmodium are responsible for one of the deadliest and most common parasite infections: malaria [167]. Insects called vectors of infected female Anopheles mosquitoes carry the disease, which affects hundreds of millions of people worldwide [150]. Hundreds of millions of individuals worldwide are afflicted by the most lethal parasitic illness [177]. Malaria still affects approximately 50% of the global population [82]. In 2020, there were expected to be 241 million cases of malaria in 85 endemic countries, up from 227 million cases in 2019, according to data from the World Health Organization (WHO) (The World Malaria Report 2021), with 95% of cases coming from Africa [203].

The WHO has developed a new statistical technique that offers more accurate estimates of the causes of all diseases, including malaria, in young children. As opposed to earlier estimates of 4.8%, the updated technique found that 7.8% of childhood mortality was related to malaria. The updated methodology showed a consistent underestimate throughout the time series and a greater than previously recognized number of estimated deaths between 2000 and 2020 [131]. It is important to note that 96% of all malaria deaths worldwide are claimed to have occurred in Africa (with > 50% of deaths occurring in Nigeria, the Democratic Republic of the Congo, Uganda, Mozambique, and Angola) [34]. The continent of Africa is where most malaria infections and fatalities occur. In 2020, sub-Saharan Africa accounted for around 95% of all cases and 96% of all malaria deaths, with approximately 80% of these deaths reported in children under the age of 5 [144].

The most at-risk groups for malaria are children under five and expectant mothers. Malaria strikes these populations hardest [201]. According to the United Nations Children and Emergency Fund, a child under the age of five dies from malaria every two minutes [19]. In 2020, there were an estimated 11.6 million (34%) pregnancies exposed to malaria infection in the World Health Organization (WHO) African Region, which resulted in 900,000 low birth weight (LBW), 25,000 maternal deaths, and 100,000 neonatal deaths [110].

Approximately 102 million people in Latin America currently reside in locations where malaria transmission poses a concern, of whom at least 28 million do so in high-risk areas (where there are more than 10 cases per 1,000 population) [44]. In the past ten years, a lot of work has been done to eradicate malaria in the Americas. The WHO claimed that Paraguay, Argentina, and El Salvador were malaria-free in the years 2018–2021, which is noteworthy [169]. Despite this, malaria is still endemic in 17 nations and territories in the region [65]. *P. vivax* causes the majority of cases reported in Venezuela (76%), compared to *P. falciparum* (17.7%) and mixed *P. vivax*/*P. falciparum* infections (6%) and *P. malariae* (1%) [77]. Currently, the Amazon Basin, which encompasses nine South American nations, is the site of the great majority of malaria cases reported [187]. This is mostly linked to deforestation, ecological changes, and extensive human movement connected to a continuing process of land occupation [102].

Plasmodium infects a variety of animals, with at least five species posing a major health danger to humans [103]. The four limited or adapted species that have historically been identified as being the causes of human malaria are *P. falciparum, P. ovale, P. malariae, and P. vivax* [38]. The parasite *P. knowlesi*, which infects simian hosts, has been recognized as the fifth most significant Plasmodium in terms of human health [105]. There are other simian malaria parasites, *P. cynomolgi* and *P. inui* in Asia and *P. brasilianum* and *P. simium* in the Americas, that have also been shown to have the potential for zoonotic transmission to humans [33, 107].

The principal causes of malaria in humans are *P. vivax* and *P. falciparum *[103]. Although *P. falciparum* is the most frequent and widely spread malaria parasite, *P. vivax* is the most prevalent, most widely disseminated, and can also cause severe life-threatening sickness [125]. This parasitic infection remains the most challenging tropical disease to eradicate [165]. *P. vivax* malaria was once thought to be a benign infection but is now recognized as a significant global health threat due to its high morbidity and mortality rates [154]. The estimated yearly burden of *P. vivax* malaria (14.3 million [13.7 to 15.0 million]) is a significant order of magnitude lower than that of *P. falciparum* (193.5 million [142.0 to 254.7 million]) [27].

The Global Technical Strategy (GTS) for Malaria was established with the objective of fully eradicating malaria by 2030 [200]. The GTS aims to achieve global case incidence and fatality rates of at least 90% by 2030 and to totally eradicate malaria in at least 35 nations [151].

According to the 2016 World Malaria Report published by the World Health Organization (WHO), the incidence rate  of malaria has fallen by 41% since 2000 [24]. Furthermore, there were no indigenous malaria cases in 23 countries for a total of three times between 2000 and 2020; 12 of these nations received the WHO certification for being malaria-free [203]. Despite these admirable accomplishments, many endemic countries still face enormous obstacles on the road to malaria eradication. The vast majority (85%) were in Africa, inflicting havoc primarily in Sub-Saharan Africa. *P. vivax* is progressively becoming the predominant source of malaria infection and disease in coendemic areas, although *P. falciparum* and *P. vivax* are the most common causes of human malaria.

### Epidemiology of *P. vivax*

*P. vivax*, one of the five malaria parasites that may infect humans, has the widest geographic distribution [207]. Geographically, 48 percent of the world's population is at risk from *P. vivax* infection. Asia, South and Central America, Oceania, the Middle East, and some regions of Africa are all affected by it, which puts 2.85 billion people in danger each year [53]. *P. vivax*, found mostly in Asia and Latin America, is possibly the most prevalent malaria parasite globally and caused 4·5 million cases worldwide in 2020 [191]. Six nations accounted for more than 85% of all *P. vivax* cases worldwide: India, Afghanistan, Pakistan, Ethiopia, Papua New Guinea, and Indonesia [71]. *P. vivax* is assumed to be the cause of 8% of estimated malaria cases globally (approximately 50% when sub-Saharan Africa is omitted), with three countries (Ethiopia, India, and Pakistan) accounting for 80% of cases [166]. *P. vivax*, as opposed to *P. falciparum*, is the primary cause of malaria in the Americas [164]. Despite the fact that *P. vivax* is an uncommon species in Africa, where *P. falciparum* predominates, it is coendemic with *P. falciparum* in Ethiopia, where case incidence rates are roughly equal. *P. vivax* is responsible for approximately 40% of all malaria infections in Ethiopia, similar to other locations, according to several lines of evidence [16, 67].

### Biology of *P. vivax*

In terms of biology, *P. vivax* and *P. falciparum* are highly distinct from one another [61]. This biological complexity helps it survive in situations that are not ideal for *P. falciparum,* allowing it to spread further [17]. This species appears to be more resistant to control efforts than *P. falciparum*, according to several control programs [88]. The therapies and monitoring methods designed for *P. falciparum* malaria may not be adequate or appropriate for *P. vivax* malaria [159]. Due to various unique aspects of *P. vivax* biology, eradication of malaria will eventually become a challenge [46].

*P. vivax* uses unique transmission strategies, such as (i) a dormant and relapsing stage in the liver (hypnozoites); (ii) reticulocyte tropism, the parasite strict reticulocyte preferences; (iii) gametocytes' rapid and consistent development; (iv) vivax malaria parasites are genetically highly variable; this genetic plasticity results in multiple clones of *P. vivax* infections, which may drive increases in parasite virulence, fitness, and survival, allowing it to respond to antimalarials differently than *P. falciparum* and other malaria parasites; (v) climatic conditions: the parasite's ability to replicate at lower temperatures than *P. falciparum*, allowing it to broaden its range far beyond the tropics; and (vi) drug resistance in the Malaria Parasite: CQ resistance appears to have spread globally [16, 16, 72, 96, 158].

In addition, many *P. vivax* vectors are early biters and outdoor feeders and exhibit outdoor-resting patterns [68]. These characteristics are supposed to make it easier to get through control measures such as insecticide-treated nets (ITNs) or indoor residual spray (IRS). As a result, *P. vivax* is less likely to be controlled by ITNs or IRS [104]. Because of this, *P. vivax* transmission would be more resilient to control measures over time than *P. falciparum*. Data from a scientific review by [115] overwhelmingly support this. Despite these findings, the WHO proposed a bold global goal of eliminating malaria in 35 countries by 2030 [200]. This goal is significantly hampered by the fact that this species has evolved. *P. vivax* must therefore have been recognized as a significant barrier to the control and eradication of malaria in coendemic areas. Table 1 summarizes the obstacles of eliminating vivax malaria as well as the implications.

**Table 1:** A summary of the obstacles and implications of eliminating & eradicating *P. vivax* malaria

Potential obstacles that could pose challenges to *P. vivax* malaria eradication and elimination	Control and eradication impacts
Hypnozoite	*P. vivax* presents a challenge for malaria management and eradication because it can develop clinically quiet, undetected, dormant liver stages that subsequently reactivate (or relapse) to generate blood-stage infections. A portion of the parasites undergo sexual differentiation during blood stage replication, and when ingested by a blood-feeding anopheline mosquito, these gametocytes can start the insect's sporogony process. Therefore, contributed substantially to both clinical episodes and transmission [25, 32]
The puzzle of primaquine therapy	Poor treatment compliance and delaying radical cure for lactating women until their nursing infants are at least 6 months old and determined to be G6PD normal and the risk of hemolysis in G6PD-deficient individuals [73, 162]
Host genetics	Genetic variation in CYP2D6, a human cytochrome P-450 isoenzyme 2D6 (CYP2D6) is crucial for the conversion of primaquine to its active metabolites and for the hypnozoite killing process in hepatocytes [124]
The younger the reticulocytes, the better for *P. vivax* infection	The predilection of *P. vivax* for reticulocytes has implications for infection dynamics, parasite reservoirs, and potential parasite elimination strategies [184]
*P. vivax* is emerging and has evolved into Duffy-negative	Shape the epidemiology of *P. vivax* malaria: previously, it was believed that *P. vivax* malaria was uncommon or nonexistent in African populations that did not exhibit the Duffy blood group antigen. Recent research has, however, documented a number of *P. vivax* infections in Duffy-negative individuals in several African regions, including those where Duffy negativity is predominated. There are also more and more instances of *P. vivax* infection among Duffy-negative people in South America, which may affect the epidemiology of *P. vivax* malaria [75, 79,112]
*P. vivax* submicroscopic	Low-density *P. vivax* infections are frequent, particularly in places that are close to elimination. Some malaria patients do not receive prompt treatment, causing the disease to spread [9]
sexual blood stages	keeps showing up early in the course of the disease, often before overt symptoms develop &increases the possibility of further transmission [6]
Temperature	Sporogony occurs at lower temperatures than for *P. falciparum* [145]
*P. vivax* populations are more genetically diverse	Wide genetic variety across parasite populations makes them more likely to evade host immune responses, complicate the development of a malaria vaccine, and possibly even reveal previously undiscovered invasion routes [94]

## Control challenges

### Hypnozoite: the forgotten obstacle

Plasmodium parasites have a complicated life cycle [192]. Infected mosquito sporozoites move to the liver and begin the hepatic stage of the parasite life cycle by penetrating hepatocytes, where they multiply and develop into schizonts containing thousands of hepatic merozoites [167]. *P. vivax*, on the other hand, has unique biological properties, including an extra stage that greatly increases the epidemiological and clinical complexity of the disease compared to *P. falciparum* [5]. That is, not all sporozoites in the liver grow into schizonts right away; some evolve into small, nonreplicating, unnucleated latent forms in the liver stage, termed hypnozoites [32]. In a study that used a mouse model, [126] found that hypnozoites exist after the primary liver stage infection has occurred. They are significantly smaller and nonreplicating than replicating liver-stage schizonts [190]. Additionally, no diagnostic tools are available at this time that can identify this stage [78]. The parasite's hypnozoite form, which may evade the human immune system and blood-stage therapy and provide a safe haven during mosquito-free seasons, can remain inside liver cells for weeks to years [147, 195]. Due to its dormancy, the parasite may endure the winter when Anopheles mosquito transmission is not possible due to the environment [46]. Eventually, the hyponozoite reactivates and multiplies, producing a blood-stage relapse and subsequent parasite transmission unless drugs specifically targeting the infection are administered [81]. Each strain may have a different ratio of hypnozoites to sporozoites, and parasites with a larger proportion of hypnozoites may be more likely to relapse frequently [46]. Additionally, the latent hypnozoite may be lying in wait when forward transmission is questionable due to conditions that do not favor the mosquito. For instance, mosquito activity tends to be seasonal in temperate climates [148]. On the other hand, *P. falciparum* causes only one attack in less than two weeks following a single contagious bite [121]. Following the initial blood-stage infection, hypnozoites activate to produce subsequent relapsing infections, and it is believed that relapses account for 79–96% of all *P. vivax* infections [92]. According to data from the Thai-Myanmar border, 3 out of 4 patients relapsed after 1441 recurrent *P. vivax* infections in 1299 patients over the course of 1000 patient follow-up years [180]. According to other data from Southeast Asia and the South Pacific, hypnozoite-induced relapses account for the majority of the burden of acute *P. vivax* infection [140]. This undetectable hypnozoite reservoir represents one of the primary barriers to malaria control since it maintains active transmission of the disease and is resistant to the existing antimalarial medications used to clear exoerythrocytic stages [15]. Furthermore, no diagnostic tools are currently available that can diagnose this stage [78]. Thus, this trait promotes the spread of parasites throughout the year and increases the difficulty of controlling the parasite.

The frequency of relapses is mostly determined by numerous hypothesized variables, including the size of the sporozoite inoculum, the host's innate immunity, the primary therapeutic regimen, coinfections, fever, hemolysis, seasonality, mosquito bites, and epigenetic debates [190]. Tropical regions have a significant (> 80%) incidence of early relapse, with subsequent relapses occurring every 3–4 weeks [159]. Additionally, the latency duration may be influenced by the sporozoite inoculation load. Each strain may have a different ratio of hypnozoites to sporozoites, and parasites with a larger proportion of hypnozoites may be more likely to relapse frequently [46].

Furthermore, because hypnozoites serve as a reservoir for a variety of *P. vivax* strains, they will supply a large number of concurrently circulating parasite clones in the bloodstream, as well as effective genetic recombination between unrelated *P. vivax* parasites [198]. *P. vivax*-infected mosquitoes can produce numerous clinical attacks attributable to hypnozoites [11]. As a result, hypnozoites make malaria control more difficult by allowing *P. vivax* parasites to spread spatially and develop drug resistance. These characteristics make the distribution of vivax in a population less responsive to vector control techniques. As a result, *P. vivax* cannot be effectively controlled using falciparum malaria control strategies.

### The primaquine issue and the puzzle of *primaquine* therapy

#### Primaquine in the real world

Primaquine (PQ), an 8-aminoquinoline, is one of the first synthetic antimalarial medicines and was discovered in 1946 [20]. Since 1952, PQ has been the sole FDA-approved treatment for the hypnozoite stage [176]. It works against *P. falciparum* mature gametocytes and the hepatic dormant stage, as well as the hypnozoites of two Plasmodium species (*P. vivax* and *P. ovale*) that can cause relapses [130]. According to a study by Cedillos RA. from El Salvador, 46–68% of individuals experienced repeated *P. vivax* episodes without receiving PQ treatment [170]. It is suspected to interfere with the parasite's oxygen consumption by creating oxygen free radicals yet disrupting the parasite's electron transport system, while the mechanism of action is unknown [35].

Despite the fact that PQ is the sole, unique, irreplaceable, and successful treatment for eradicating *P. vivax* hypnozoites, it is also accompanied by substantial risks and side effects [52]. Side effects such as gastrointestinal disturbances (nausea, dizziness, and vomiting), hypersensitivity reactions, and life-threatening severe hematological adverse effects, particularly in patients with inborn erythrocytic G6PDd (methemoglobinemia and hemolytic anemia), have limited the drug's utility [26, 31]. There are several PQ regimens in common use today. The currently WHO recommended treatment, people with *P. vivax* malaria should be treated with chloroquine for three days to kill the parasites that cause malaria symptoms in the blood, then 0.25 mg/kg of body weight (in a single daily dose) of PQ for 14 days to treat hypnozoite-derived relapses ('radical cure') of vivax malaria [118]. Based on a study conducted in Brazil by [50], the total dose given influences the radical curative efficacy so that compliance is essential for a radical treatment because effectiveness is correlated with the total PQ dose given. One of the clinical drawbacks of this treatment regimen is that it is more than four times longer than conventional schizontocidal regimens, which limits adherence to the lengthy regimen [118, 162]. Nonadherence to this PQ treatment varies from 2 to 40% [111]. Patients typically experience symptom improvement within a few days of beginning CQ, hence, they frequently do not adhere to the PQ treatment plan's 14-day duration [8]. Lack of an immediate advantage, a lack of knowledge of the long-term benefits for the individual and society, and the misconception that vivax malaria is a benign disease are other reasons that contribute to nonadherence [111].

On the other hand, for the radical cure of *P. vivax*, PQ 0.5 mg/day for 7 days is employed; this shorter, higher dose regimen is being used in several nations, such as Brazil [135]. The same total dose (0.5 mg/kg/day to 210 mg) administered over 7 days as opposed to 14 days may have little to no difference on *P. vivax* recurrences [127]. Several surveys showed that despite the short duration, less than 70% of people completed the short-course primaquine regimen. This may be because of its complicated treatment regimen, which still makes adherence difficult, and its occasional subpar effectiveness [136]. Even though research from South America suggests that a PQ dose of 0.5 mg/kg for 7 days is safe and well tolerated, overweight people have a greater rate of relapses caused by subtherapeutic PQ doses [118].

A significant portion of malaria patients are excluded from PQ use in pregnant and nursing mothers, leaving them partially cured and still susceptible to *P. vivax* relapses [45]. As a result, many women who breastfeed for an extended period of time are prohibited from receiving radical therapy. Contraindications to PQ usage in pregnant and breastfeeding mothers exclude a major number of malaria patients who are subsequently not fully cured and are nonetheless vulnerable to *P. vivax* relapses [73]. As a result, many women who continue to breastfeed for a long time are banned from obtaining radical treatment. Therefore, malaria relapses during pregnancy may result in congenital malaria, and radical treatment of *P. vivax* in this group is still challenging [46]. PQ resistance is sometimes confused with treatment failure, even though genuine resistance to PQ has not been demonstrated by independent sources (relapse incidence) [8]. In a recent study conducted in Ethiopia, individuals who had 14-day PQ had a 17% higher probability of developing *P. vivax* parasitaemia again [3]. The WHO goal of eliminating malaria by 2030 could therefore be jeopardized by the aforementioned variables if *P. vivax* transmission could be sustained.

To circumvent this roadblock, the possible inclusion of the hypnozoitocidal drug tafenoquine (TQ) (Krintafel) was approved by the Food and Drug Administration as a single-dose regimen to treat patients with confirmed *P. vivax* infection for radical cure [92]. Despite this benefit, hemolysis of the earlier erythrocytes continues in individuals with G6PD deficiency until all susceptible cells have been destroyed. This is because the drug's delayed clearance (terminal half-life of approximately 15 days) makes single-dose treatment possible [101]. This orally active, 8-aminoquinoline drug is eliminated much slower than PQ (14 to 28 days versus 4 to 6 h) [76]. Tafenoquine solves the potentially important problem of poor adherence to daily primaquine by providing a radical cure in a single dose [85]. In contrast, due to primaquine's quick elimination (4–9 h), stopping the drug can significantly reduce drug-induced hemolysis [176].

#### Hemolysis caused by PQ and TQ in G6PD deficiency

The most important danger of 8-aminoquinolines is dose-dependent hemolysis in people with G6PD deficiency [197]. G6PD is an enzyme that serves as a housekeeper in all cells and protects red blood cells from stress [186]. People who live in nations where malaria is endemic are more likely to have G6PDd. In fact, the frequency is significantly higher (8%) in countries with a high malaria infection rate [29]. G6PD deficiency can be frequent in these groups. Data showed that this X-linked abnormality is very common, affecting almost 400 million individuals worldwide [113].

In persons with G6PD deficiency, both drugs, PQ and TQ can hemolyze red blood cells, resulting in anemia [98]. PQ's hemolytic activity has long been thought to be caused by intraerythrocytic oxidative stress mediated by redox-active metabolites rather than the parent drug. Splenic macrophages are considered to recognize oxidatively damaged RBCs as the equivalent of senescent red cells, resulting in their removal from circulation [74]. On the other hand, TQ, as a single-dose medication, eliminates a significant drawback of the 7- or 14-day primaquine regimen: the possibility of poor adherence. However, this benefit comes with a significant risk of hemolytic toxicity with G6PD deficiency, too, because once tafenoquine is administered, it cannot be "stopped" if there is drug-induced hemolysis [111].

Both PQ and TQ use are made challenging by the lack of a quick G6PD test or the availability of a standard G6PD test. This problem could hinder efforts to eradicate malaria [60, 109]. Both PQ and TQ radical treatment have the potential to completely transform the management and eradication of vivax malaria, but to ensure its safe delivery, it will be required to create the requisite instruments for assessing G6PD status [55]. Testing for G6PD is currently offered in a variety of ways. Flow cytometry and spectrophotometry are the gold standard tests. Despite the fact that these tests measure enzyme activity, they are expensive and require an efficient lab infrastructure [109]. The most recent WHO treatment guidelines state that it is an excellent practice to use a patient's G6PD status to guide primaquine administration. Recently, more emphasis has been placed on safe primaquine medication, guided by testing for G6PD status before prescription. It is comforting to know that quick, point-of-care (PoC) G6PD test kits are available [99].

#### Genetic variation in CYP2D6 and PQ metabolism: PQ Pharmacogenomics

Numerous drugs are metabolized by an isoenzyme family called cytochrome P450 (CYP), which is mostly found in the endoplasmic reticulum of liver cells [84]. About 25% of all medications used in clinical practice are metabolized by the cytochrome P450 2D6 (CYP2D6) enzyme [119]. The drug-metabolizing enzyme Cytochrome P450 2D6 (CYP2D6) is produced by the CYP2D6 gene [152]. This human cytochrome P-450 isoenzyme 2D6 (CYP2D6) is essential for the conversion of primaquine to its active metabolites and for the hypnozoite killing procedure in hepatocytes [122]. CYP2D6 also contributes to the partial metabolization of TQ [124].

This gene is unique in that it exhibits a wide range of variants, ranging from complete inactivity (poor metabolizers) to manifold augmentation, which have a significant impact on the performance of the enzyme [194]. Among the variations in CYP2D6 (CNV), these are caused by single-nucleotide variants (SNVs), whole-gene deletions, small insertions and deletions, multiplications, tandem arrangements, and changes in gene copy number (CNV) [124, 179]. As a result, the CYP2D6 genetic polymorphism, which is very variable, may have an impact on the pharmacokinetics of primaquine [172]. False PQ tolerance assumptions in the parasite result from people with specific CYP2D6 polymorphism alleles being unable to metabolize PQ and possibly failing treatment [50]. It has been demonstrated in both animal models and people that primaquine's capacity to metabolize to its active metabolite is decreased by decreased CYP2D6 activity [156]. It influences plasma concentrations of PQ and its metabolites and is connected to PQ therapy failure in *P. vivax* malaria, as demonstrated in both animal models and humans As proven in both animal models and humans, it affects plasma concentrations of PQ and its metabolites and is linked to PQ treatment failure in *P. vivax* malaria [174].

Eradication attempts targeting PQ use could encounter significant difficulties due to the varied nature of CYP2D6 activity, as many groups worldwide, especially those in endemic locations, have a high prevalence of CYP2D6 impairments [171]. In Indonesia, directly monitored high-dose primaquine treatments resulted in 95% of therapeutic failures, according to research by [178] Therefore, it is crucial to establish methods to prevent relapse in individuals who are unable to undergo PQ treatment. In addition to safety issues, drug compliance, the ideal dose depending on bodyweight, and the follow-up duration need to be examined.

### *Plasmodium vivax* tropism to reticulocytes and Duffy antigens

#### Bone marrow reticulocytes: the younger, the better?

RBC age appears to be a significant restriction for malaria parasites [42]. Reticulocytes are a diverse population of red blood cell precursors with ribonucleic acid residues that represent the final stage of erythropoiesis before full maturation into red blood cells (RBCs) [173]. Reduced expression of the transferrin receptor CD71 (TfR1 or CD71) indicates reticulocyte maturation [185]. *P. vivax* has a narrower cell tropism than previously assumed, infecting all reticulocytes but restricted preferences only to young reticulocytes with high transferrin receptor CD71 (TfR1 or CD71) [108]. This was well investigated by [120] from *P. vivax* isolated in Thailand. *P. vivax* preferentially invades very immature reticulocytes expressing high levels of the transferrin receptor CD71 on their surface. Immature CD71 + reticulocytes are most commonly found in the bone marrow, where they are created, reside, and are generally confined [184]. Due to the low proportion of reticulocytes (0.5–1%) among all cells in human blood, *P. vivax* parasitemia is maintained at low levels [80]. The high number of submicroscopic and/or asymptomatic infections caused by reticulocyte tropism and the low parasitemia levels that accompany reticulocyte tropism are undetected by field tests such as microscopy or fast diagnostic tests (RDTs) [10, 114]. These asymptomatic infections remain untreated and may contribute to transmission over several weeks or months [63].

This phenomenon raises the intriguing possibility that *P. vivax* biomass occurs in extravascular tissues of the marrow and spleen rather than in circulating blood [168]. Recent work has also demonstrated that a substantial proportion of the biomass of asexual *P. vivax* trophozoites and schizonts occurs in the extravascular spaces of marrow, spleen, and liver [11]. Consequently, parasite densities in the blood are often low and undetectable, creating significant challenges for the diagnosis and treatment of infected individuals. The dynamics of infection, parasite reservoirs, and putative parasite killing mechanisms are all impacted by *P. vivax's* tight affinity for young reticulocytes, making malaria elimination problematic.

#### Infection of Duffy-negative erythrocytes by *P. vivax*: a recent adaptation

*P. vivax* malaria was previously thought to be uncommon or nonexistent in African populations that did not express the Duffy blood group antigen. This remains an out-of-date viewpoint [83]. However, numerous instances of *P. vivax* infection in patients who tested negative for Duffy have recently been documented in a number of African nations, including Angola, Benin, Botswana, Cameroon, Ethiopia, Equatorial Guinea, Kenya, Madagascar, Mali, Mauritania, Senegal, Sudan, and Uganda [79]. Among Duffy-negative people in Africa, 24 (88.9%) experienced *P. vivax* infections, according to a meta-analysis by [204]. These results disprove the notion that *P. vivax* infection is completely prevented by erythrocytes lacking the Duffy antigen receptor for chemokines (DARC) [79]. This action raises important issues concerning how *P. vivax* enters the erythrocytes of Duffy-negative individuals. It has been hypothesized that copy number variation, which is either low expression of DARC in Duffy-negative individuals, binds easily to parasites that carry multiple copies of *P. vivax* Duffy binding protein (PvDBP), changes in PvDBP1 or duplication in the PvDBP gene created a new entryway and is responsible for the parasites' increased ability to spread [97, 155]. Due to this, it is challenging to control and eradicate *P. vivax* malaria, which highlights the concern that these 'new' *P. vivax* strains that infect Duffy-negative hosts could spread throughout much of Africa and have severe, significant effects on the general public health and economy.

In addition, a study from Ethiopia by [1] revealed that patients with *P. vivax* infection who had the Duffy-negative genotype displayed a consistently low asexual parasitaemia (median, 53 parasites/L). This low asexual parasitaemia in Duffy-negative patients may be an "undetected silent reservoir," which would undoubtedly make it more challenging to eradicate vivax malaria [21]. Additionally, this may make it more difficult to comprehend the epidemiology of vivax malaria in the area.

### The tip of the iceberg: *P. vivax* submicroscopic

Considering that *P. vivax* invasion is entirely erythrocyte age specific, the rigorous requirement for young reticulocytes has implications for malaria diagnosis [95]. This is because *P. vivax* invades reticulocytes, which make up a minor portion of the circulating erythrocytes, and *P. vivax* infections are frequently misdiagnosed because parasitaemia is too low [75]. Duffy-binding protein (DBP) and host reticulocyte-binding protein (HRBP) appear to be required for erythrocyte invasion by the *P. vivax* merozoite (RBP) [86]. One of the *P. vivax* erythrocyte binding proteins (pvRBPs) and EBPs is thought to be important in reticulocyte recognition, particularly in young (CD71^high^) reticulocytes. DARC is not implicated, however, because reticulocytes and mature red cells (normocytes) both express identical amounts of DARC on their surfaces [40]. Because parasites also appear to "sequester" in the bone marrow [141], the number of *P. vivax* blood stages circulating in peripheral blood may not provide a realistic indication of the total parasite biomass retained by the host. One of the pvRBPs and EBPs is thought to be important in reticulocyte recognition, particularly in young (CD71high) reticulocytes [40]. These findings help to explain why *P. vivax* parasitemias are naturally and consistently lower than *P. falciparum* parasitemias [173]. For identical infection rates, the proportion of infections detected by microscopy appears to be similar for *P. falciparum* and *P. vivax*. However, because parasite prevalence rates for *P. vivax* in communities are often lower than those for *P. falciparum*, the proportion of missed infections may be larger overall for *P. vivax* than for *P. falciparum* [200].

*P. vivax* parasite counts at clinical presentation are typically 4 000 ± 3 000 parasites per liter of blood (p/L), which is three to four times lower than *P. falciparum*, and peak parasitemia seldom surpasses 100 000 p/L in *P. vivax* but is relatively common in *P. falciparum*. In low-prevalence locations, submicroscopic infections appear to be of greater relative relevance, posing an additional barrier to eradication attempts [54].

Furthermore, a positive parasitological test result from either microscopy or a rapid diagnostic test (RDT) is required before treatment can begin, according to WHO criteria for malaria diagnosis and treatment [157]. According to a review [90], 69.5 percent of all *P. vivax* blood-stage infections are submicroscopic, and asymptomatic *P. vivax* causes 89–100 percent of submicroscopic infections. Furthermore, according to a Brazilian cohort study [70], microscopy missed 4 percent of *P. vivax* infections discovered by polymerase chain reaction (PCR); 57 percent of them caused no clinical signs or symptoms indicative of malaria, and 33 percent were both subpatent and asymptomatic. As a result, some malaria patients do not receive prompt treatment, causing the disease to spread. Malaria diagnosis therfore requires sensitive and economical diagnostics for detecting low-load infections and infections in all population carriers who may or may not display clinical symptoms to accomplish elimination [134].

### *P. vivax* cytoadhering: immune evasion occurrence

RBCs infected with *P. falciparum* have the capacity to cytoadhere to several host cell types, including endothelial cells and red blood cells that are not infected, and sequester in the microvasculature [175]. Due to its ability to stay in deep vascular beds and avoid being removed by the spleen, this occurrence is essential to the parasite's immune-evasion strategy [58, 106]. *P. vivax's* inability to cytoadhere has long been thought to exist. However, investigations have demonstrated that *P. vivax*-infected erythrocytes (Pv-iE) can cytoadhere to host cells in vitro [62].

Mature *P. vivax-*infected erythrocytes (*Pv*-IEs) have been demonstrated to cytoadhere to human lung endothelial cells, Saimiri brain endothelial cells, and cryosections of the placenta in earlier in vitro studies from Manaus (Brazil) [39]. The database for adhesion characteristics in *P. vivax* parasites is now expanding [188]. Carvalho and associates first showed that PvIRs can cling to human lung endothelial cells (HLECs), Saimiri brain endothelial cells (SBECs), placental cryosections, CSA, and the cell-surface receptor ICAM-1 in a study by [161] According to Brazilian research [123], rosetting was observed in 64% of the isolates, CSA adherence in 15%, ICAM1 adhesion in 12%, and placental cryosections in 9%. Additionally, the findings of a study by [206] show that *P. vivax*-infected red blood cells (PvIRBCs) rosette irreversibly with normocytes and are significantly stiffer than nonrosetting PvIRBCs. Further evidence reveals that *P. vivax* can, to some extent, exhibit pathogenic profiles comparable to *P. falciparum* given the presence of severe types of malaria in *P. vivax* infections, such as cerebral malaria and placental malaria, which were previously reported to be exclusively linked with *P. falciparum* [188].

### Transmission and early development of sexual blood stages (gametocytes)

Gametocytes from *P. falciparum* and *P. vivax* have highly different biology [137]. These Plasmodium species have different maturation times for gametocytes [49]. Within a few days of the first appearance of the asexual stage, *P. vivax* gametocytes swiftly appeared in the bloodstream [100]. This differs significantly from *P. falciparum*, whose gametocytes mature one week later [23]. Because sequestration in tissues is not a key phase in *P. vivax* development, gametocytes mature significantly faster than *P. falciparum* gametocytes [100]. Evidence also suggests that *P. vivax* forms gametocytes at a rate that is higher (up to 20% every cycle) than that of *P. falciparum* [149]. Mature *P. vivax* gametocytes are found in the bloodstream far earlier and before the beginning of clinical illness, virtually certainly before patients seek treatment [142].

The relatively quick gametocyte development of *P. vivax*, which occurs concurrently with asexual parasite stages, as opposed to 10–12 days in *P. falciparum*, accounts for the early transmissibility [143]. This shorter embryonic cycle of *P. vivax* is thought to increase the likelihood of mosquito infections with various genotypes, resulting in more recombination among those genetically heterogeneous parasites [162]. This indicates that *P. vivax* will be more transmissible than *P. falciparum* because mosquito infections are more efficient [159]. The majority of gametocyte carriers were also asymptomatic, which suggests that silent infections may play a key role in the spread of malaria. This has been amply established in Thailand, where a significant percentage of *P. vivax* gametocytes are found in asymptomatic infections [138], indicating a potentially significant contribution to the transmission reservoir. As a result, the human parasite reservoir comprises asymptomatic *P. vivax* infections in people who do not seek medical help. This reveals that *P. vivax* gametocytes are a transmission control bottleneck [189]. When compared to *P. falciparum, P. vivax* transmission is more likely to be stable over time, undermining control efforts. As a result, the *P. vivax* malaria control strategies established for falciparum malaria will not be as effective [146].

### Genetic diversity

Genetic diversity is one of the most important strategies by which the malaria parasite may maintain a long-term infection despite a continual immune response. *P. vivax* populations show higher genetic variation than *P. falciparum* populations across their genome [53]. Evolutionary fitness is provided by genetic diversity, which increases the possibility for adaptation to changing circumstances [128]. As a result, parasite populations with a wide genetic diversity are more likely to resist antimalarial host immunological responses [57]. Furthermore, the presence of distinct parasite forms in different geographic regions, as well as their diverse genotypes, presents challenges to the creation of a malaria vaccine [36, 207].

*P. vivax* has exhibited considerable genomic diversity in several population genetic studies based on microsatellite data and, more recently, whole genomes [18]. Furthermore, investigations have revealed that the genome of *P. vivax* parasites circulating in the same location is more diverse than that of *P. falciparum* parasites [53]. The parasite clones that coexist in natural infections are frequently genetically diverse. Reactivating genetically varied hypnozoites causes more frequent outcrossing during meiotic recombination and a quicker creation of new parasite strains, which increases the genetic complexity of blood-stage infections [64].

Antigenic variation, repeat-number variation in microsatellites, gene copy-number variation (CNV), and single-nucleotide polymorphisms (SNPs) have all been discovered in isolates from various geographic origins [48, 53]. Garzón-Ospina et al. [72] observed that such polymorphisms are often present in functionally irrelevant genes. This could open up new invasion paths that were previously unknown.

This high amount of antigenic polymorphism shows the presence of sophisticated mechanisms that have allowed this parasite species to escape and overcome host immune responses [193]. Because many critical immunological targets and vaccination candidates display significant polymorphism, high antigenic diversity poses a significant challenge when developing a vaccine [41]. That is perhaps why only limited candidate vaccines are believed to have entered Phase I clinical trials, compared to 23 *P. falciparum* vaccine candidates [13, 182]. According to another study by [116], sequence diversity was seen in gene families linked to immune evasion and erythrocyte invasion, suggesting that vaccines targeting polymorphic antigens may face an even greater challenge in eliciting an effective immune response than they do in *P. falciparum*, where strain-specific immunity has been shown to limit vaccine efficacy.

Furthermore, superinfection by genotypes that are unrelated can result in many parasite clones [208]. During their life cycles, these strains interact, and these interactions can result in intricate patterns of interspecies exchanges and intrahost competition [2]. Drug-resistant *P. vivax* strains are becoming more common, which could be linked to parasite genetic diversity [91]. According to [160], *P. vivax* is growing increasingly resistant to chloroquine, the first-line treatment, and is refractory to most types of antimalarial medicines. Furthermore, the reactivation of genetically varied *P. vivax* hypnozoites increases the genetic diversity of blood-stage infections by allowing for more frequent outcrossing during meiotic recombination and the formation of novel parasite strains [12].

Overall, genetic diversity assures evolutionary fitness, boosting the possibility of adapting to changing environmental conditions. Additionally, drug resistance is more likely to develop in parasite populations with a range of genetic make-ups [94]. In general, *P. vivax's* genetic diversity may help the parasite adapt to new challenges, such as improved treatments and control strategies.

### Climate conditions: temperature

Climate factors, such as temperature, rainfall patterns, and humidity, have a significant impact on the life cycles and survival of parasites and vectors, which in turn greatly affects the susceptibility to transmission of diseases such as malaria [69]. The biology of parasites is heavily influenced by temperature [117]. The main reason why malaria is frequently referred to as a climate-dependent disease is that mosquito sporozoites require a specific range of temperatures to fully mature [196]. The amount of temperature variation that occurs throughout the day has an impact on site development [28]. An experimental report found that the *P. falciparum* sporogony in mosquito vectors is temperature-sensitive. When compared to *P. falciparum*, *P. vivax* has a lower minimum temperature at which sporogonic development takes place [27]. *P. vivax,* on the other hand, can tolerate a wider range of environmental temperatures than the more virulent *P. falciparum* (minimum: 16 °C vs. 21 °C for *P. falciparum*), which may help to explain why it is more distributed widely and exhibits very efficient transmission rates across a wider range of climates [38]. Unlike other human malaria species, *P. vivax* grows, although slowly, at lower temperatures to develop in the vector, between 16 and 18 °C, whereas *P. falciparum* can only grow at temperatures higher than 18 °C [129]. *P. vivax* sporogony in the vector is shorter (~ 10 days at 25 °C) than for *P. falciparum* (12 days) [153]. Due to its sporozoites' ability to mature at lower temperatures, *P. vivax* is temperature agnostic [158]. This form of development dramatically widens the parasite's worldwide range, allowing it to spread more widely across the globe and the parasite's ability to establish long-term transmission foci in temperate climates rather than only in tropical climates [200].

Furthermore, several major *P. vivax* vectors have specific characteristics, including early biting, outdoor feeding, and outdoor resting [25]. There are large rates of early and outdoor transmission of vivax malaria, according to data from various regions. According to data from western Eritrea, 36.4 percent of infective bites were acquired outside, while up to 49 percent of outdoor transmission in Uganda occurred before bedtime [59]. Thus, the two main vector control strategies, ITNs and IRS, are not necessarily as efficient against *P. vivax* as they are against *P. falciparum* in terms of reducing mosquito life duration by targeting indoor and nocturnal biting mosquitoes.

Implementing an environmental alteration known as "species sanitation" is one method for controlling vectors and eliminating *P. vivax *[15]. This method provides prevention without relying on the numerous issues and difficulties associated with diagnosis and treatment or the limitations of insecticidal methods.

### Drug Resistance in the Malaria Parasite: A Threat to the Eradication of Malaria

It is very concerning that antimalarial drug resistance is steadily growing and spreading [30]. Additionally, it puts attempts to manage and eradicate malaria at risk [133]. *P. vivax* is currently known to be resistant to antimalarial drugs [87]. Additionally, pharmacological interactions that lead to cross-resistance between drugs with the same chemical family or similar modes of action can also be blamed for the worsening of antimalarial drug resistance [37]. There have been numerous reports of *P. vivax* resistance to chloroquine in endemic regions [43].

CQ resistance appears to have spread globally, based on the genetics of *P. vivax* [93] Numerous chloroquine and antifolate resistance-related mutations were discovered in *P. vivax* samples, including SNP and Pvcrt-o K10-insertion combinations that may indicate chloroquine-resistant *P. vivax* phenotypes, according to research from southern Thai provinces [139]. Additionally, due to the widespread incidence of *P. vivax* that is resistant to chloroquine, certain countries have been forced to transition from chloroquine to artemisinin-based combination therapy (ACTs), which has an effect on the use of primaquine as the only antirelapse drug [56, 66]. These include Papua New Guinea, the Solomon Islands, Sudan, Namibia, South Africa, and Vanuatu [22]. Because Plasmodium has developed drug resistance to the available antimalarial drugs, managing and eliminating malaria has thus become increasingly challenging. The evaluation of antimalarial efficacy offers a potential solution to this issue by lowering the chance of failure brought on by parasite resistance to certain therapies.

Despite the fact that primaquine resistance is sometimes mistaken for therapy failure or the inability to eradicate the *P. vivax* hypnozoite liver stage following the completion of the complete course of medication and the appropriate therapeutic dose [183], primaquine and chloroquine resistance in *P. vivax* has been well reported [16]. Primaquine treatment failure has been documented in several *P. vivax* strains, especially those from the Western Pacific, Southeast Asia, South America, and certain regions of Africa [199]. A case report from Ethiopia by [181] shows the failure of primaquine for the treatment of relapsed *P. vivax* malaria.

### RTS, S/AS01 vaccine for falciparum but not for vivax malaria

There is currently no *P. vivax* vaccine that is widely available, and there will not be any very soon [51]. Children in sub-Saharan Africa and other areas with moderate to high *P. falciparum* malaria transmission are recommended to receive the malaria vaccine RTS,S/AS01 (RTS,S), which mimics protein-coated infectious sporozoites [205]. Other forms of malaria, such as *P. vivax*, are not protected against by vaccination. In contrast, the protective antibody responses against malaria sporozoites elicited by RTS,S rely on the neutralizing action of antibodies existing at the time of sporozoite infection [205].

The recommendation is based on the outcomes of a pilot program that has been running in Ghana, Kenya, and Malawi since 2019 and has reached more than 900,000 children [202]. The majority of children and adults will carry parasites that will infect mosquitoes, although because this vaccine does not confer extensive sterile immunity and RTS, S-induced immune responses do not interfere with the infectivity of gametocytes (the transmission stages of Plasmodium). As a result, transmission will not change, maintaining endemicity [205]. The suggested *P. falciparum* vaccine is ineffective against *P. vivax* and other types of malaria [14]. Adopting RTS,S would also provide indirect benefits, such as a decrease in malaria-related all-cause hospitalization, which would free up possibly limited health resource allocations for other people in need [47].

## Alternative approaches to *P*. *vivax* elimination: Tafenoquine for radical cure and implementation of Rapid point-of-care diagnostics for G6PD

The malaria community as a whole recognizes that early diagnosis and treatment are essential to eliminating malaria. The US Food and Drug Administration (FDA, July 2018) and the Australian Therapeutic Goods Administration (TGA), two regulatory bodies, have officially approved the 8-aminoquinoline derivative tafenoquine (TQ) as a revolutionary treatment for *P. vivax* malaria [89]. As seen by more recent relative increases in the *P. vivax/P. falciparum* ratios in many coendemic countries, this method has been less successful in controlling *P. vivax than P. falciparum*. The main causes of this disparity are *P. vivax's* tendency for relapse and the lack of therapies that are both secure and efficient for the hypnozoite reservoirs. Two recent occurrences show that radical cure and, thus, hypnozoite eradication will be more commonly available. The first is TQ, an 8-aminoquinoline with a long half-life that has been licensed and is being implemented in endemic countries. TQ may provide an alternative to the current treatment options. The ideal alternative would have the following qualities: a shorter treatment period; greater efficacy in hypnozoite clearance; absence of PQ's important side effects, including hemolysis in those with G6PD deficiency; and absence of variance in the parasite's susceptibility (partially contributed by host genetic polymorphisms) [163]. The second also included the introduction of point of care. Rapid point-of-care diagnostics for G6PD are now available, which would make it possible to test for the disease in distant areas and avoid the need to transport patients to more advanced medical facilities for extreme measures.

## Conclusions and future directions

Control measures such as mass drug administration, screening, vaccine development, vector control, and treatment campaigns could not affect *P. vivax*, unlike *P. falciparum*. In addition, due to the lack of an in vitro continuous culture that maintains the parasite erythrocytic cycle, *P. vivax* has received less study attention than *P. falciparum*, which has become a major hurdle. Overall, no genuine chance of eradication is predicted in the next few years unless new tools for lowering malaria transmission are developed (i.e., malaria elimination and eradication).

Therefore, what is next for malaria caused by *P. vivax* control and elimination? What will be the parasite's future defense strategies?

The study of Plasmodium parasites will benefit from systematic methodologies, which will lead to a major improvement in the fight against malaria. By utilizing further genome-wide sequencing tools, our understanding of Plasmodium biology is expanded, and new study routes are beginning to appear (such as "omics").

The "omics" field has the potential to clarify a number of biological problems. This field enables us to comprehend Plasmodium spp. biology, in particular the dynamics of RNA and protein expression and regulation throughout its complex, multistage life cycle, in host and vector interaction contexts and under varied environmental selective pressures. Comprehensive knowledge about cellular components and biomolecules, such as genes (genomics & epigenomics), RNA (transcriptomics), proteins (proteomics), and metabolites (metabolomics), will be provided by these "omics" methods to biological network dynamics [7].

1. Genomes & epigenomes:—A significant area of research in Plasmodium genome biology is the identification of genes evolving under selective forces favoring novel alleles or sustaining diversity within populations. Epigenomes to understand transcriptional regulation. Whole genome sequencing (WGS) enables a more thorough examination of an organism's genetic make-up and the discovery of protective antigens. The availability of primary Plasmodium genome sequences has also improved our knowledge of the host and parasite elements that contribute to infection and enabled the identification of potential treatment targets by combining them with animal models of infection to comprehend transcriptional control and use epigenomes.
2. Studies of *P. vivax* transcriptomes**:** transcriptomes to determine mRNA steady state may offer special insights into the biology of this parasite and its distinctions from *P. falciparum*. Furthermore, a study by Muller et al. [132] found that a number of transcripts implicated in the early infection of the vertebrate host are not immediately recognized as proteins and may be subject to translational repression.
3. Proteomes of parasite particles and parasite-secreted proteins in plasma, according to [7], create significant amounts of parasite proteins that can be utilized for diagnostic purposes. Proteomics may also examine proteomes and subproteomes simultaneously without needing any prior knowledge of the types of proteins. Large quantities of parasite proteins that can be exploited for diagnosis are produced by parasite particles and parasite-secreted proteins in plasma proteomics. Proteomics also offers a substantial advantage in the search for new target biomolecules since it can concurrently analyze proteomes and subproteomes without needing to know the type of proteins involved. There is also much potential in the study of protein expression, interactions, and alterations. Additionally, [4]’s research revealed 153 proteins from the *P. vivax* blood stages. A startling discovery was that more than 36% of the parasite proteome was made up of hypothetical proteins.
4. Using interactomes & Metabolomics’: to comprehend how protein‒protein interactions work, and with the development of metabolomics, it is now able to examine metabolites more easily and predict the chloroquine resistance of infected patients by finding altered pathways and significantly altered metabolites. These studies will offer a crucial direction for the eradication and extinction of malaria. Furthermore, molecular biology knowledge will support the development of rapid diagnostic tests (RDTs) for diagnosis, drug development, monitoring of drug resistance, and improved research into the manufacture of malaria vaccines.

In summary, it is quite likely that new methods for eliminating vivax malaria will be needed, necessitating the creation of high-value products, including vaccinations that prevent transmission, fresh drug combinations to treat chloroquine-resistant strains, and a secure, long-lasting 8-aminoquinoline.

## Literature search strategy

This review article was created using published research on *P. vivax* malaria and malaria in general. Searches were conducted in online public databases such PubMed, Google Scholar, ScienceDirect, Web of Science, and other relevant journals that published reviews on *P. vivax* to locate published articles for the review. *P. vivax*-related malaria is researched and documented in these databases. The original research articles and review papers used in all of the studies included in the systematic review were published in English. The duplicate papers were not considered in the systematic review. EndNote version X8 was used to generate the arrangement.

## Data Availability

Not applicable for review work.
